# Stent-in-stent deployment for malignant hilar obstruction using multi-hole stent can prevent tumor ingrowth during 6-month cholangioscopic follow-up

**DOI:** 10.1055/a-2518-5430

**Published:** 2025-02-05

**Authors:** Takeshi Ogura, Yuki Uba, Takafumi Kanadani, Kimi Bessho, Hiroki Nishikawa

**Affiliations:** 1 Endoscopy Center, Osaka Medical and Pharmaceutical University Hospital, Takatsuki, Japan; 2 2nd Department of Internal Medicine, Osaka Medical and Pharmaceutical University, Takatsuki, Japan

Malignant hilar biliary obstruction (MHBO) can be treated by bilateral uncovered self-expandable metal stent (SEMS) deployment using a stent-in-stent (SIS) technique 1 . Recently, patient survival has been prolonged as a result of recent improvements in systemic chemotherapy, including immune checkpoint inhibitors; therefore, prolonged stent patency is necessary. As there is a risk of tumor ingrowth with an uncovered SEMS, stent patency might not be good. A fully covered SEMS (FCSEMS) can prevent tumor ingrowth, and stent patency might therefore be longer than with an uncovered SEMS. However, because an FCSEMS can obstruct a bile duct branch, FCSEMS deployment for MHBO using the SIS technique may not be feasible. Recently, an FCSEMS with side holes (HANAROSTENT Biliary Multi-hole NEO; M.I. Tech Co., Ltd, Pyeongtaek, South Korea) has become available (MHSEMS) ( Fig. 1 ). This stent was designed to prevent stent migration through small tissue ingrowths that form in the multiple small (1.8 mm) side holes along the covering membrane 2 3 . In addition, through this hole, SIS deployment can be performed ( Fig. 2 ). We present a case of successful SIS deployment using the new MHSEMS and describe cholangioscopic findings on long-term follow-up.

**Fig. 1:**
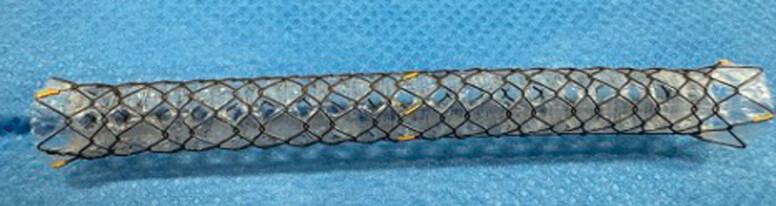
The fully covered self-expandable metal stent with side holes (HANAROSTENT Biliary Multi-hole NEO; M.I. Tech Co., Ltd, Pyeongtaek, South Korea).

**Fig. 2:**
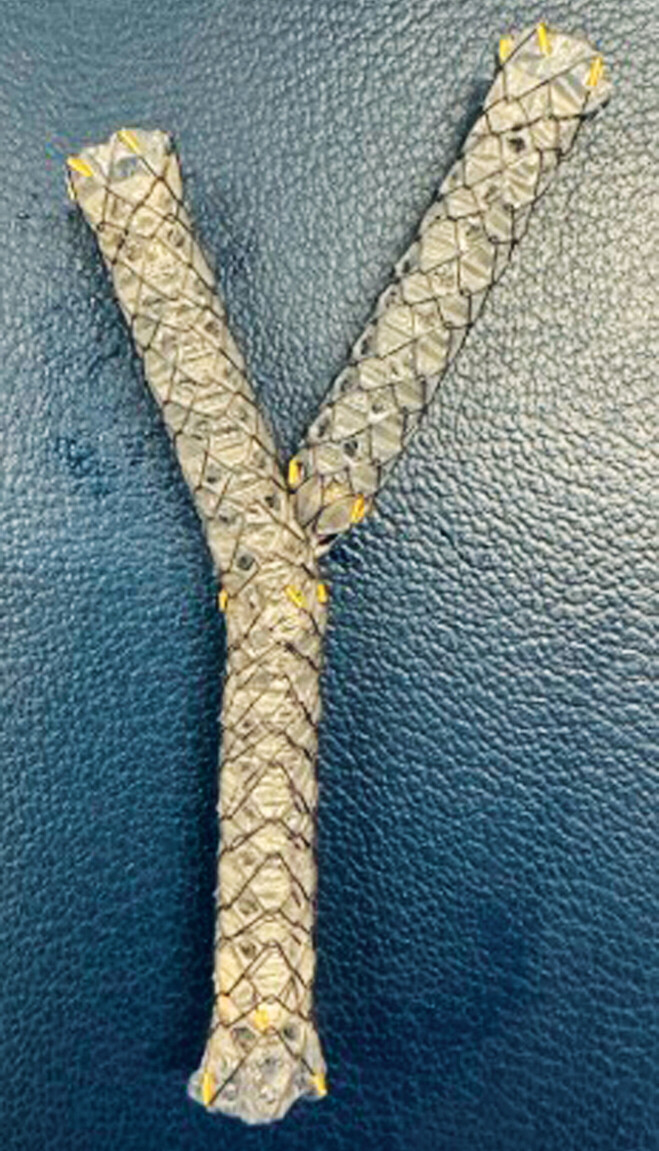
The fully covered self-expandable metal stent with side holes was designed to prevent stent migration through small tissue ingrowths that form in the multiple small (1.8 mm) side holes along the covering membrane; stent-in-stent deployment can also be performed through these holes.

A 55-year-old man was admitted to our hospital due to unresectable MHBO. Before systemic chemotherapy, biliary drainage was attempted. After successful biliary cannulation and contrast medium injection, bile duct obstruction was observed. First, the stent delivery system for an MHSEMS was inserted into the left bile duct and successfully deployed. Next, the guidewire was inserted into the right hepatic bile duct. Finally, an MHSEMS was deployed at the right hepatic bile duct using the SIS technique ( Fig. 3 ).

**Fig. 3:**
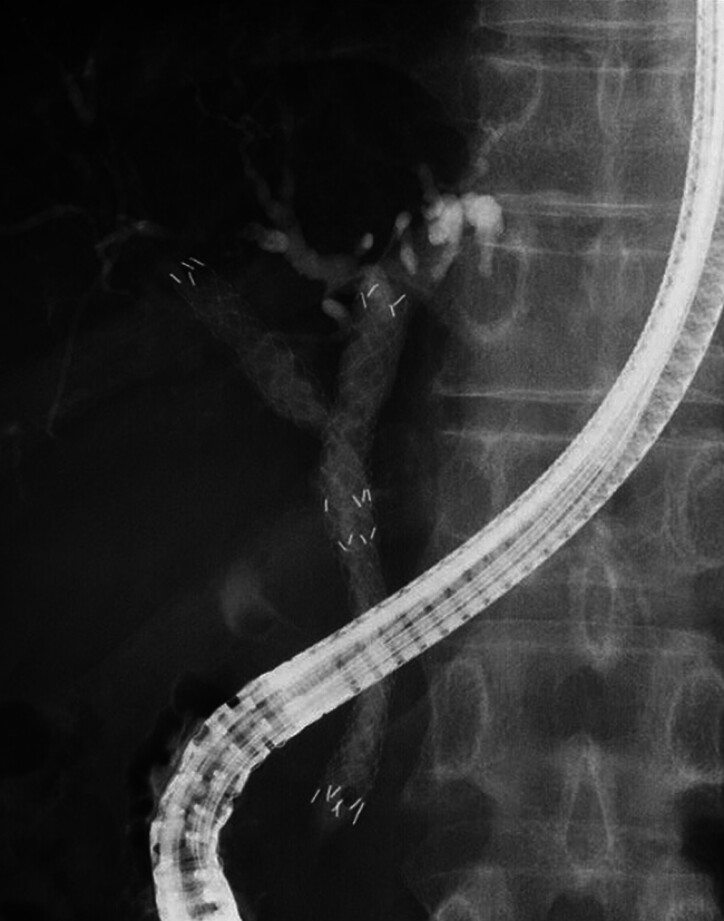
A fully covered self-expandable metal stent with side holes was deployed at the right hepatic bile duct using the stent-in-stent technique.

Recurrent biliary obstruction was seen 6 months later. An endoscopic retrograde cholangiopancreatography catheter was inserted into the stents, and contrast medium was injected. Although neither bile stent was obstructed, lower bile duct stenosis was observed. A cholangioscope was inserted to evaluate the condition of the stents. Tumor ingrowth had been completely prevented ( Fig. 4 ), but lower bile duct obstruction was observed and considered to be due to lateral tumor spread ( Fig. 5 , Video 1 ). Therefore, an FCSEMS was deployed for the lower biliary stricture. After this reintervention, recurrent biliary obstruction was not observed until the patient’s death (4 months later).

**Fig. 4:**
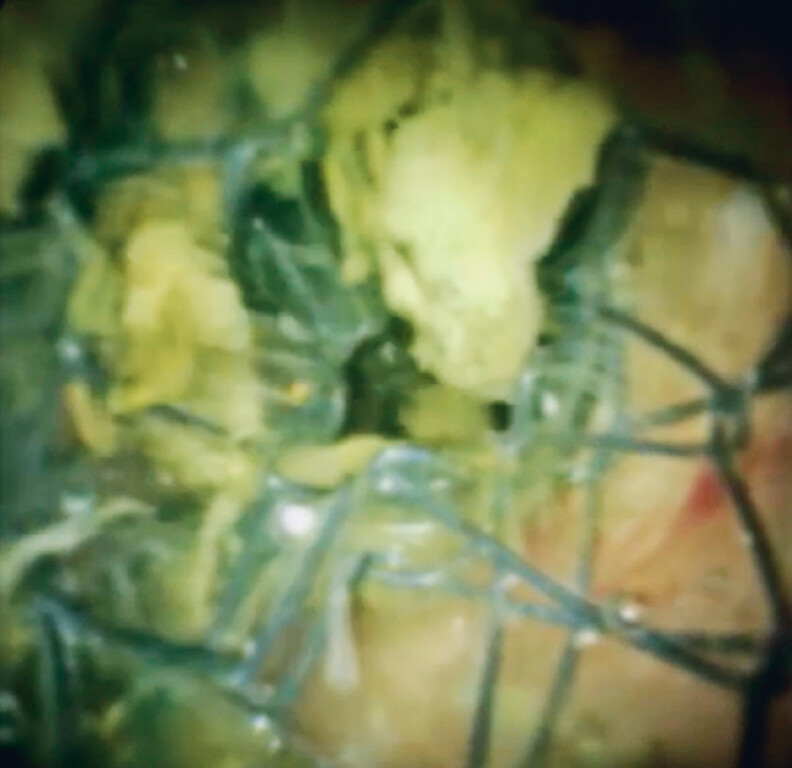
Tumor ingrowth was completely prevented.

**Fig. 5:**
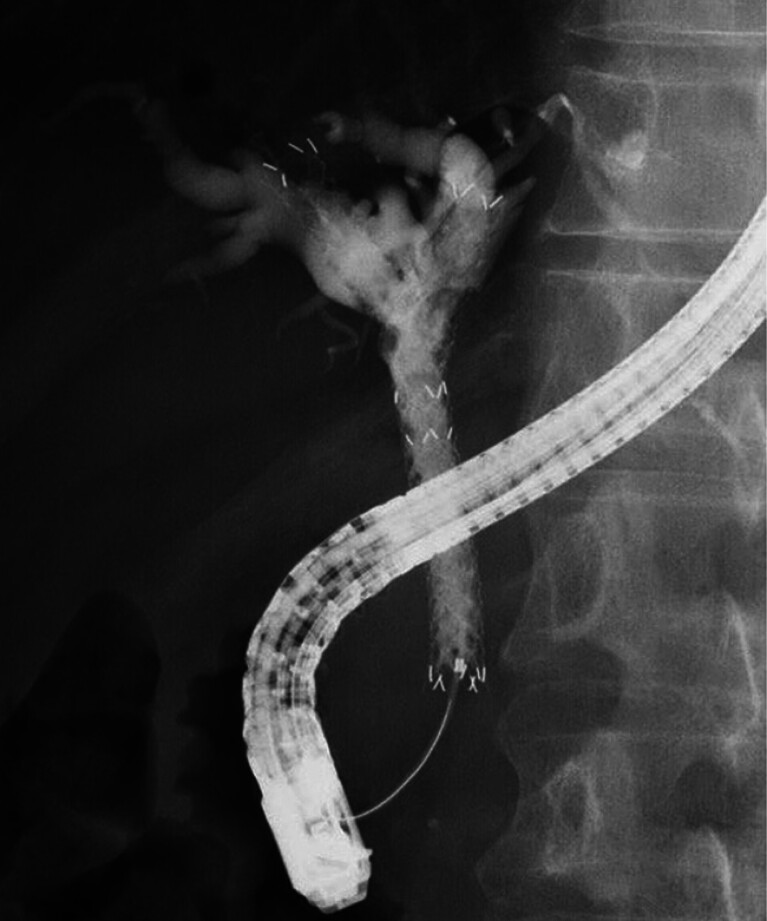
Lower bile duct obstruction was observed and considered to be due to lateral tumor spread.

Bile duct obstruction by the fully covered self-expandable metal stent with side holes was not observed.Video 1

In conclusion, the SIS technique using an MHSEMS for MHBO may achieve longer stent patency by preventing tumor ingrowth. A randomized trial comparing uncovered SEMS and MHSEMS is needed.

Endoscopy_UCTN_Code_TTT_1AR_2AZ

## Notes

Endoscopy E-Videos https://eref.thieme.de/e-videos
*E-Videos* is an open access online section of the journal *Endoscopy* , reporting on interesting cases and new techniques in gastroenterological endoscopy. All papers include a high-quality video and are published with a Creative Commons CC-BY license. Endoscopy E-Videos qualify for HINARI discounts and waivers and eligibility is automatically checked during the submission process. We grant 100% waivers to articles whose corresponding authors are based in Group A countries and 50% waivers to those who are based in Group B countries as classified by Research4Life (see: https://www.research4life.org/access/eligibility/ ). This section has its own submission website at https://mc.manuscriptcentral.com/e-videos .
